# Case Report: Bispecific CD20/CD30-targeted chimeric antigen receptor T-cell therapy for non-Hodgkin’s lymphoma

**DOI:** 10.3389/fimmu.2025.1567149

**Published:** 2025-05-08

**Authors:** Yuejiao Huang, Yiming Gong, Xiang Liu, Huaying Ruan, Jinhua Lu, Hosein Kouros-Mehr, Hong Liu, Han Wang

**Affiliations:** ^1^ Department of Hematology, Affiliated Hospital of Nantong University, Nantong, China; ^2^ TriArm Therapeutics (Shanghai), Shanghai, China; ^3^ Shanghai First Song Therapeutics, Shanghai, China; ^4^ TriArm Inc., San Mateo, CA, United States; ^5^ Department of General Surgery, Affiliated Hospital of Nantong University, Nantong, China

**Keywords:** CAR T-cell therapy, transformed follicular lymphoma, CD20, CD30, bispecific CAR T

## Abstract

CD19-directed CAR T-cell therapy is a breakthrough immunotherapy for B-cell malignancies. However, CD19 loss-mediated relapsed/refractory disease continues to pose a significant challenge, highlighting the urgent need for CAR T cells targeting alternative antigens. To address this issue, we developed a CD20-directed CAR T incorporated with an additional CD30-directed binder to enhance cytotoxicity toward cancer cells. Here, we report that a patient with bulky transformed follicular lymphoma was successfully treated with CD20/CD30-directed CAR-T cells. The patient received two doses of anti-CD20/CD30-CAR-T therapy administered one month apart. Complete metabolic remission was achieved 1 month after the first infusion without evidence of cytokine release syndrome (CRS) or immune effector cell-associated neurotoxicity syndrome (ICANS). The second dose was given as a consolidation therapy with sustained disease-free survival exceeding 12 months to date. The report underscores the promising therapeutic potential and safety profile of CD20/CD30-directed CAR T-cell therapy.

**Clinical Trial Registration:**

https://www.clinicaltrials.gov, identifier NCT06756321.

## Introduction

Chimeric antigen receptor (CAR) T-cell therapy has transformed the clinical landscape of hematologic malignancies. Currently, FDA has approved 5 CAR T-cell products for the management of B-cell malignancies, demonstrating remarkable efficacy with an overall remission of 71-81% in B-cell acute lymphoblastic leukemia (1–3), and 52-97% in B-cell non-Hodgkin lymphoma (B-NHL) (4–10). Despite the high initial response rate, relapsed or refractory disease following CAR T still represents a challenge, with subsequent salvage treatments providing less than 3 months of progression-free survival benefit (11). The loss of CD19 antigen, which is the target of all 5 commercial products, was reported as one of the major contributors to disease relapse (12–16), emphasizing the need for alternative targets to be explored.

CD20 is a B cell marker universally expressed on the surface of normal B cells and most mature B-cell malignancies. For several years, CD20-targeted therapy involving monoclonal antibodies and T cell engagers have served as the cornerstone in the management of B-NHL. In clinical trials, CD20-targeted CAR T-cell therapies have exhibited potent anti-lymphoid effect in various B-NHL cancers (17–22), particularly in cases of relapsed or refractory disease following CD19-directed CAR T treatment (17, 22). In one study, anti-CD20 CAR T-cell therapy generated a complete remission rate of 57.1% even after failure of anti-CD19 CAR-T treatment (17), which is comparable to the efficacy in CAR T-naïve NHLs (4, 7, 9). The above studies suggested that CD20 could be a promising target for CAR T development.

CD30 is mostly found to express in a subset of activated lymphocytes and lymphomas, including classical Hodgkin lymphoma (cHL), anaplastic large-cell lymphoma (ALCL) (23, 24). Brentuximab Vedotin (BV), a CD30-targeted antibody drug conjugate, has been approved for the treatment and maintenance of cHL, and for combo therapy with lenalidomide and rituximab against large B-cell lymphoma. Given its success in various lymphoid malignancies, CD30 has emerged as a promising target for CAR T-cell therapy. Most anti-CD30-CAR T-cell studies have demonstrated remarkable efficacy against CD30^+^ lymphomas, with objective response rates ranging from 37.5% to 91.7% (25–29). Notably, Kochenderfer et al. reported a 43% transient anti-lymphoid response in CD30^+^ lymphomas, with extensive rash and prolonged hematologic toxicities (30). Despite these challenges, anti-CD30-CAR-T have shown clinical responses in some CD30^+^ lymphomas, solidifying CD30 as a compelling target for further CAR T-cell development.

Multi-specific CAR T is a potential strategy to enhance the potency of CAR T-cell therapies. By targeting 2 or more antigens on tumor cells, tumor microenvironment (TME), or immune cells, multi-specific CAR design can potentially prevent antigen escape-mediated relapse (12, 31–36), remodel the tumor microenvironment (37–39), boost CAR T-cell expansion (40), and enhance CAR T-cell function (41). In 2019, Abken et al. reported that co-targeting of CD30 in CEA- and TAG72-targeted CAR T cells enhanced T cell activity against CD30-negative tumor cells via elimination of CD30^+^ T cells, which suppress the cytotoxic T cell response. Moreover, CD30 is expressed in 10-30% of NHL cancers (42–44). Beyond the direct activity against CD30^+^ NHL, BV combined therapy with rituximab, a CD20-targeted agents, produce robust clinical response in large B-cell lymphoma regardless of CD30 expression (45, 46), suggesting a potential synergistic antitumor effects with CD20/CD30 dual targeting therapies. The dual-targeting strategy may improve therapeutic outcomes and reduce the risk of relapse due to antigen escape.

Therefore, we developed a CAR T-cell product targeting both CD20 and CD30, for patients with relapsed/refractory B-cell non-Hodgkin lymphoma, including patients who progressed following conventional CD19-targeted CAR T cells. Here we report a case using the CD20/CD30 bispecific CAR T to treat a bulky transformed follicular lymphoma.

## Case description

**Figure 1:**
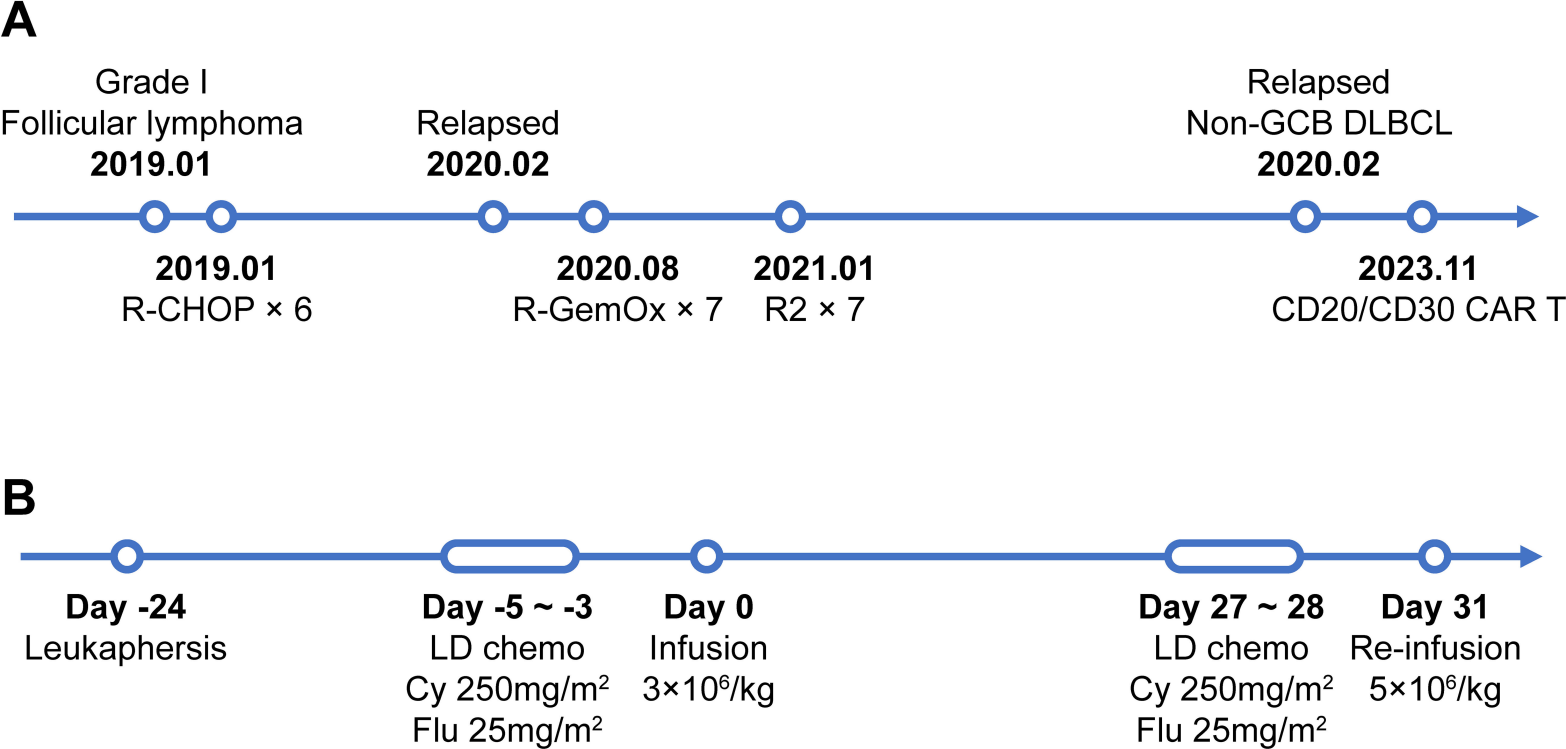
The timeline of patients’ treatment and trial intervention. **(A)** Flow chart of the disease process and therapeutic modalities before CAR T treatment. **(B)** The timeline of CAR T preparation and administration from leukapheresis to the 2^nd^ dose of CAR T infusion. Abbreviations: Non-GCB DLBCL, Non-germinal center diffuse large B cell lymphoma; LD chemo, lymphodepleting chemotherapy; Cy, cyclophasphomide; Flu, fludarabine.

A 60-year-old female patient with extensive lymphadenopathy (cervical, supraclavicular and abdominal nodes) was diagnosed with grade 1 follicular lymphoma (stage III) in January 2019 (**Figure 1A**). She was first treated with 6 cycles rituximab combined with cyclophosphamide, pirarubicin, vincristine, and prednisone (R-CHOP) and then obtained a partial response by CT-scan. As the patient had no symptoms at the time, she refused further treatment.

The first relapse occurred approximately 3 months after the last chemotherapy. She developed right inguinal lymphadenopathy, and further core needle aspiration revealed a B cell lymphoma with the following immunohistochemistry (IHC) results: CD20+ (3+), CD30-, Bcl-2+ (3+), Bcl-6+ (3+), Ki-67+ (40%+), and MUM1-. She was subsequently treated with R-GemOx (consisting of rituximab, gemcitabine and oxaliplatin). Two months into her treatment, her abdominal lymph nodes have significantly reduced in size, but there has been little change in the lesions in other areas. Unfortunately, PET-CT scans after 7 cycles R-GemOx demonstrated progressive disease with enlarged lymph nodes and increased FDG uptake (Deauville 5) in left cervical, left supraclavicular, left axilla, para-aortic, presacral, left obturator, right inguinal, and left gluteal intermuscular area. The treatment was then switched to R-DHAP (Rituximab, Dexamethasone, High-dose Cytarabine, Cisplatin) but was discontinued due to intolerance. Rituximab combined with lenalidomide (R2 regimen) was then administered for 5 cycles. No further treatment or evaluation was performed.

Approximately 1.5 years later, the patient was admitted with left gluteal pain and limited physical activity for 7 months. Her MRI scan showed an occupying lesion (73×66 mm) in the left gluteus maximus and partial mid-arm muscle area. The third pathological investigation revealed non-germinal center B cell like (GCB) diffuse large B cell lymphoma (DLBCL) with invasion of striated muscle tissue, positive for Bcl-2 (90%+), Bcl-6 (60%+), CD20 (3+), Ki67 (80%+), MUM1 (80%+) and c-myc (50%+). PET-CT showed a bulky mass (68×64×101mm) in the left buttocks with increased SUV uptake (SUVmax=30) and an enlarged left inguinal lymph node with slightly increased SUV uptake (SUVmax=5.6). Similarly, the extremely high LDH concentration (>3 × upper limit of normal) also reflected an extensive tumor burden. In light of the patient’s history of FL, this DLBCL was diagnosed as a transformed lymphoma.

Due to ineffective treatment approaches, the patient visited the affiliated hospital of Nantong University in search of a CAR T-cell therapy. Immunohistochemistry (IHC) analysis revealed diffuse positivity for CD20, partial positivity (80%) for CD19, and scattered/weak positivity for CD30 in the tumor cells (**Supplementary Figure S1**). Following a comprehensive discussion of available therapies, the patient opted to participate in an investigator-initiated clinical trial of tandem anti-CD20/CD30 CAR T-cell therapy (NCT No.: NCT06756321) instead of pursuing CD19-directed CAR T-cell therapy. Leukapheresis was successfully performed and peripheral blood mononuclear cells (PBMCs) were collected for manufacturing the 3rd generation tandem anti-CD20/CD30 CAR T-cells (The CAR construct and characterization is given in **Supplementary Figure S2**). After a mild lymphodepleting chemotherapy (cyclophosphamide 250mg/m^2^ d1-d3, fludarabine 25mg/m^2^ d1-d3), a total dose of 1.7×10^8^ CAR T-cells (3×10^6^/kg) were then infused (**Figure 1B**).

**Figure 2:**
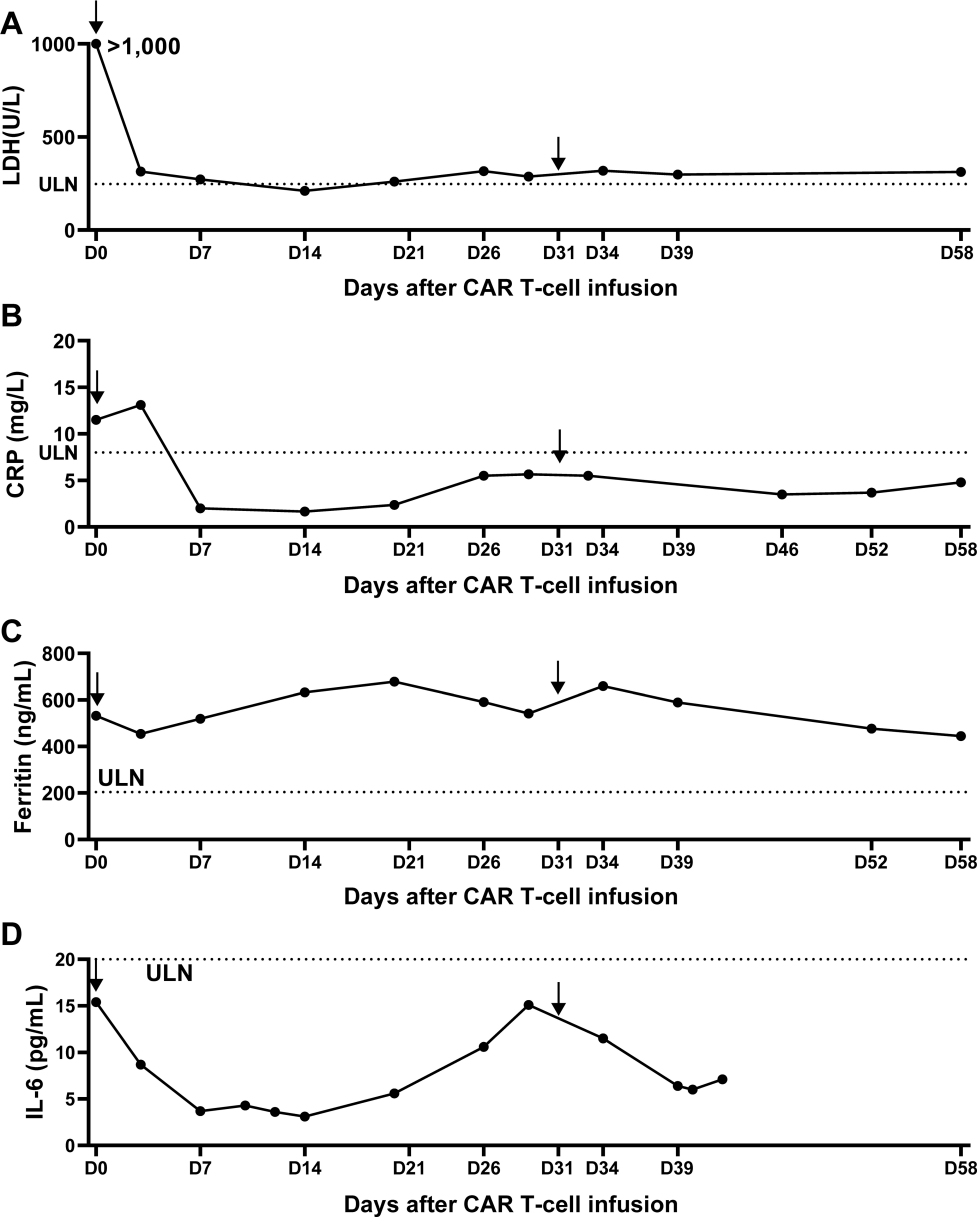
Measurement of lactate dehydrogenase and CRS-related indicators over 2 months after anti-CD20/CD30-CAR-T infusion. **(A)** The serum lactate dehydrogenase (LDH) was remarkably decreased after CAR T infusion. **(B-D)** Serum C-reactive protein **(B)**, serum ferritin **(C)** and IL-6 **(D)** showed no significant changes over the first 2 months after CAR T infusion, consistent with the absence of CRS. Arrow indicates the timepoint of CAR T infusion and the doted lines indicate the lower limits of normal ranges.

**Figure 3:**
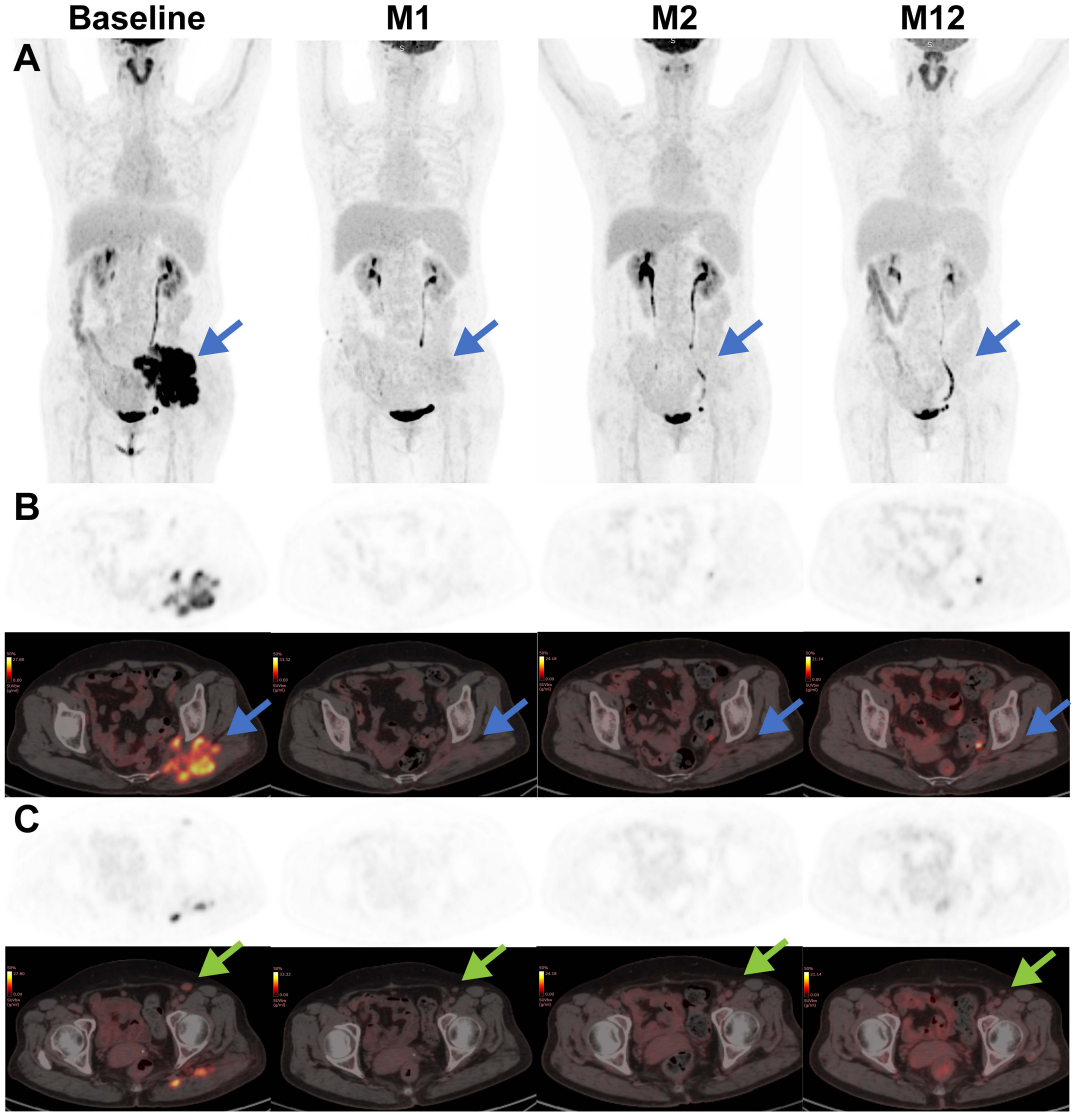
Representative coronal-view and axial-view images of serial 18F-FDG PET/CT scans before and after CAR T treatment. The baseline PET-CT shows a bulky mass (blue arrow) in the left buttocks with SUVmax of 30 and an enlarged left inguinal lymph node (green arrow) with SUVmax of 5.6. After CAR T infusion, the lymphoma lesions showed a rapid decrease in metabolic activity and a slow reduction in size, supporting the conclusion of complete remission. **(A)** Maximum intensity projection PET images; **(B)** axial images of lymphoma lesion in the left buttocks; **(C)** axial images of enlarged left inguinal lymph node.

Following the infusion, her left gluteal pain was soon relieved and no longer affected her daily life. In parallel, the serum LDH remarkably decreased and returned to normal range within 2 weeks (**Figure 2A**). At month 1 after infusion, PET-CT showed a clear regression of FDG uptake in the left buttocks and inguinal lymph node (**Figure 3**). Notably, the patient did not experience cytokine release syndrome (CRS) or neurotoxicity or notable changes in inflammatory markers or cytokines (**Figures 2B–D**) despite a rapid therapeutic response. Except hematologic toxicity (Grade 4 lymphocytopenia and grade 3 neutropenia), no other adverse events were observed.

**Figure 4:**
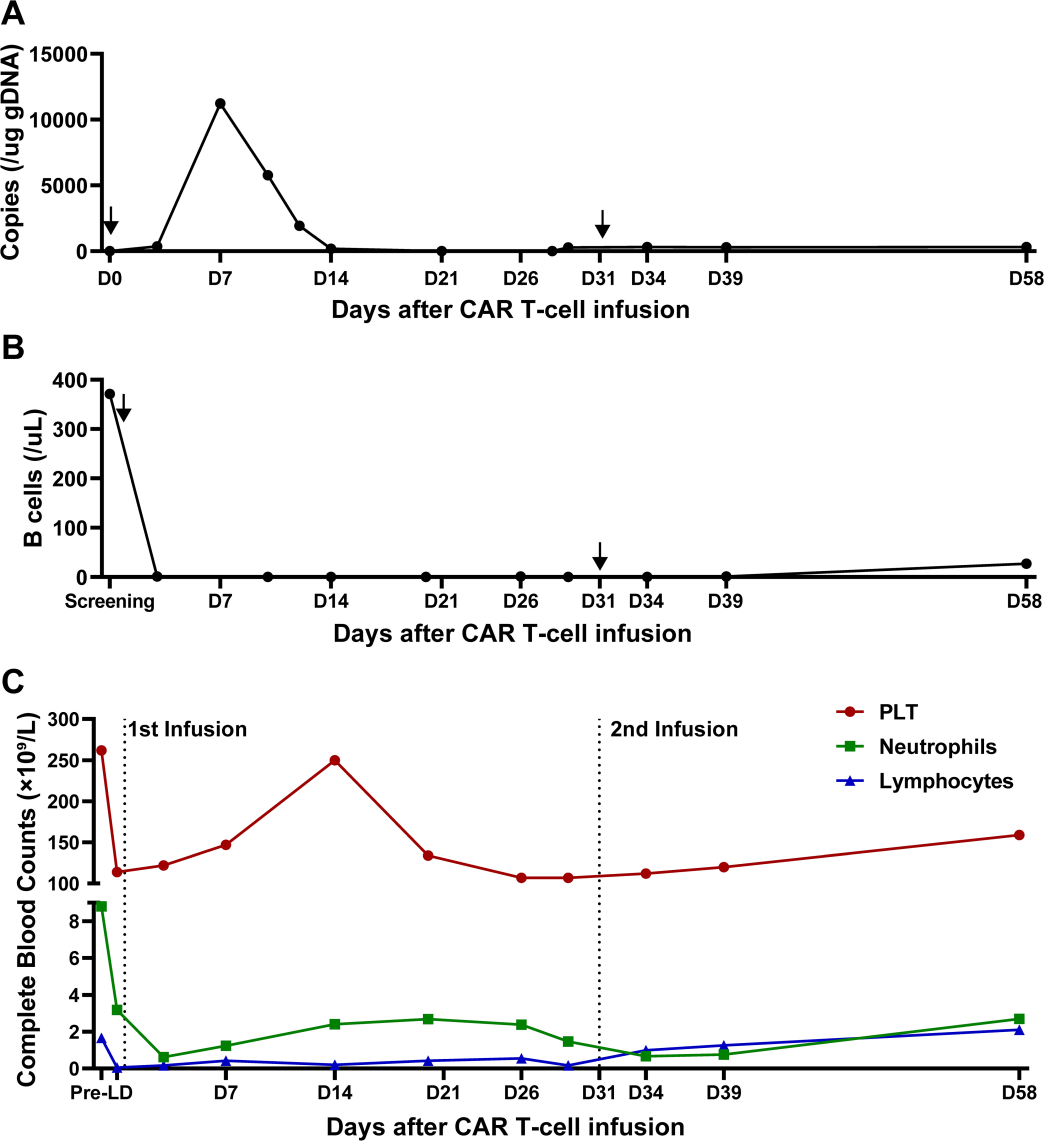
CAR T cells and other blood cells dynamics after infusion. **(A)** Quantification of anti-CD20/CD30 CAR copies detected by qPCR after infusion. Arrows indicate the timepoint of CAR T infusion. **(B)** B cell reconstitution after CAR T infusion. Data is shown as B cell counts detected by flow cytometry in peripheral blood. Arrows indicate the timepoint of CAR T infusion. **(C)** The recovery of platelet counts, absolute neutrophil counts and absolute lymphocyte counts during CAR T treatment. Dot lines indicate the timepoint of CAR T infusion.

After T cell infusion, CAR T expansion was analyzed at the indicated time points via quantitative polymerase chain reaction (qPCR). As demonstrated in **Figure 4A**, CAR T-cells began to expand *in vivo* after 3 days, peaked on day 7 and then gradually decreased, with a Cmax of 11,228.9 CAR copies/μg DNA. Meanwhile, circulating B cells remained at extremely low levels after infusion.

At month 1, the patient experienced complete metabolic response and had hypometabolic lesions (Deaville score 3). Considering that circulating CAR T cells were no longer detectable at month 1 and the patient still experienced mild edema in the left gluteus maximus (with the possibility of residual tumor), an additional 2.8×10^8^ CAR T-cells (5×10^6^/kg) were given as a consolidative treatment following a reduced-dose LD regimen (cyclophosphamide 250mg/m^2^ d1-d2, fludarabine 25mg/m^2^ d1-d2) (**Figure 1B**). Although the patient developed mild fever and fatigue after reinfusion, the 2nd CAR T failed to engraft with minimal levels of CAR copies detected in the peripheral blood (**Figure 4A**). B cells, neutrophils, and lymphocytes began to rebound 1 month after the 2nd CAR T re-infusion (**Figures 4B, C**). At month 2, the patient continued to have complete metabolic response which was maintained for approximately 12 months following initial CAR T infusion (**Figure 3**).

## Discussion

Here we report the treatment of a relapsed/refractory transformed follicular lymphoma patient with anti-CD20/CD30 bispecific CAR T-cells. The treatment was well tolerated with no CRS or ICANS and expected hematologic toxicities. Peak levels of CAR-T cells occurred on day 7 following CAR T-cell infusion and the patient reached CMR at 1 month, which was maintained for over 12 months following infusion.

In this case, while the 1^st^ CAR T infusion expanded significantly *in vivo*, the 2^nd^ consolidative CAR T cells appeared to fail to engraft. While re-infusion often exhibiting poor expansion and suboptimal efficacy (47, 48), CAR T-cell expansion is in general related with various factors, including target antigen burden and CAR design. Following the 1^st^ CAR T dosed, the patient experienced rapid resolution of symptoms, accompanied by normalization of LDH levels within 2 weeks. The PET-CT scan confirmed a complete metabolic response at Month 1. The absence of target antigen may explain both the failed expansion of the 2^nd^ CAR T dose and the rapid disappearance of the 1^st^ CAR T cells from Day 14 to Month 1. Furthermore, CD30 has been reported to be expressed on activated T cells (24). The introduction of an additional anti-CD30 CAR might have induced fratricide during late-phase of CAR T expansion, contributing to their poor persistence. Notably, the suboptimal CAR T persistence did not compromise tumor control in this case - a finding seemingly contradictory to the conventional paradigm that durable tumor control requires long-term CAR T persistence. Further studies may provide more insights on expansion and persistence of anti-CD20-CD30 CAR T cells vs clinical responses.

It has been well-established that CD30 is a biomarker of classic Hodgkin lymphoma (cHL) (49). In contrast, CD30 expression can be detected at various frequencies in NHL, including primary mediastinal B-cell lymphoma (PMBCL) (50) and mediastinal gray-zone lymphoma (MGZL) (51). BV monotherapy in NHL has been disappointing. However, the combination of BV and rituximab plus lenalidomide had a significant improvement in overall survival compared with rituximab and lenalidomide plus placebo in patients with relapsed and refractory DLBCL (45, 46). The overall survival benefit suggests potential synergy of CD20- and CD30-targeted treatment in NHL. The quick metabolic response in our case after bispecific anti-CD20-CD30 CAR T cell infusion seems consistent with this hypothesis.

Bispecific CAR T has several advantages over monospecific CAR-T. First, bispecific anti-CD20 and -CD30 CAR-T can be administered to patients with NHL and HL because expression of CD20 and CD30 can be detected in both diseases. Second, multi-specific CAR-T may potentially mitigate antigen escape mediated tumor relapse (52, 53), which is frequently observed after monospecific CAR T treatment in clinical trials. However, multi-targeting CAR-T may also have its drawbacks. The clinical efficacy of dual-targeting CAR T-cell therapy has been inconsistent. Several studies have reported that CD19/CD22 dual-targeting CAR T cells exhibit non-superior objective response rates and shorter response durations in ALL or LBCL, potentially due to inadequate CAR T-cell persistence or pharmacokinetics (12, 54, 55). Furthermore, multi-targeting CAR T-cell may potentially result in the loss of multiple targetable antigens (12, 56, 57), thereby restricting further therapeutic options. Another concern is that conventional dual-targeting CAR T strategies often rely on co-transduction with separate viral vectors, which may lead to large burden of vector integration and increase genotoxicity concerns. To mitigate these risks, our study utilizes a tandem CAR design delivered via a single vector, substantially minimizing potential safety issues.

A caveat of the study is that the patient did not receive a CD19-targeted therapy before CAR T-cell infusion. Although CD19 CAR-T has been approved for the treatment of NHL, up to 30% patients relapse due to antigen loss, mutation, or other reasons. We aim to investigate the efficacy of anti-CD20/CD30 bispecific CAR T-cells in patients who relapse after CD19 CAR T therapy. We also plan to evaluate escalating doses of bispecific CD20/CD30 CAR T-cells in future.

## Conclusion remarks

This case report demonstrates the safety and efficacy of CD20/CD30 CAR-T cell therapy in the treatment of transformed follicular lymphoma. Our results provide evidence that bispecific CAR T-cell therapy may be a promising strategy for relapsed and refractory B-cell malignancy. Further research efforts will focus on potential efficacy in patients with heterogenous expression of CD20 and CD30 and with prior CD19-directed CAR T-cell treatment.

## Data Availability

The original contributions presented in the study are included in the article/**Supplementary Material**. Further inquiries can be directed to the corresponding authors.
