# Supplementary material for: Case Report: Bispecific CD20/CD30-targeted chimeric antigen receptor T-cell therapy for non-Hodgkin’s lymphoma
Source: Front Immunol. 2025 May 8;16:1567149. doi: 10.3389/fimmu.2025.1567149 (PMC12096171; doi:10.3389/fimmu.2025.1567149)
Supplement: Supplementary file 1 [file DataSheet1.docx]

Supplementary Materials

# Supplementary Figure 1


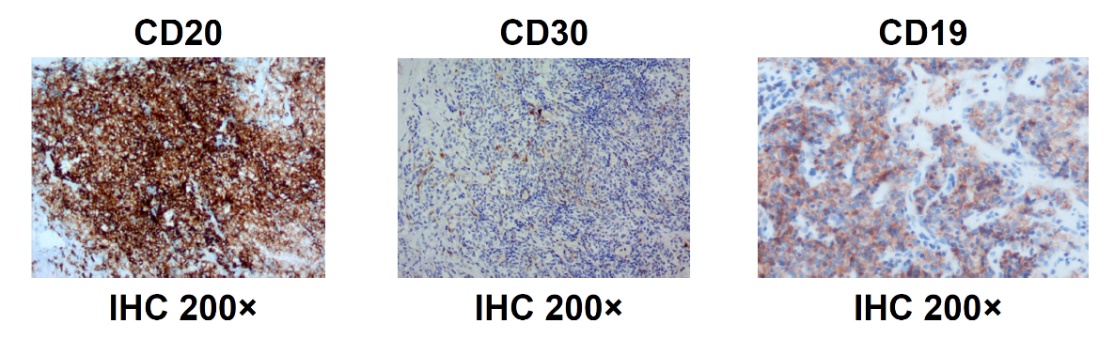


Immunohistochemistry staining of CD20, CD30 and CD19 on lymphoma tissues. Tumor cells show diffuse strong expression of CD20, scatter/weak expression of CD30 (15%+, 2%++). 80% of the tumor cells are positive for CD19. Magnification, 200×.

# Supplementary Figure 2


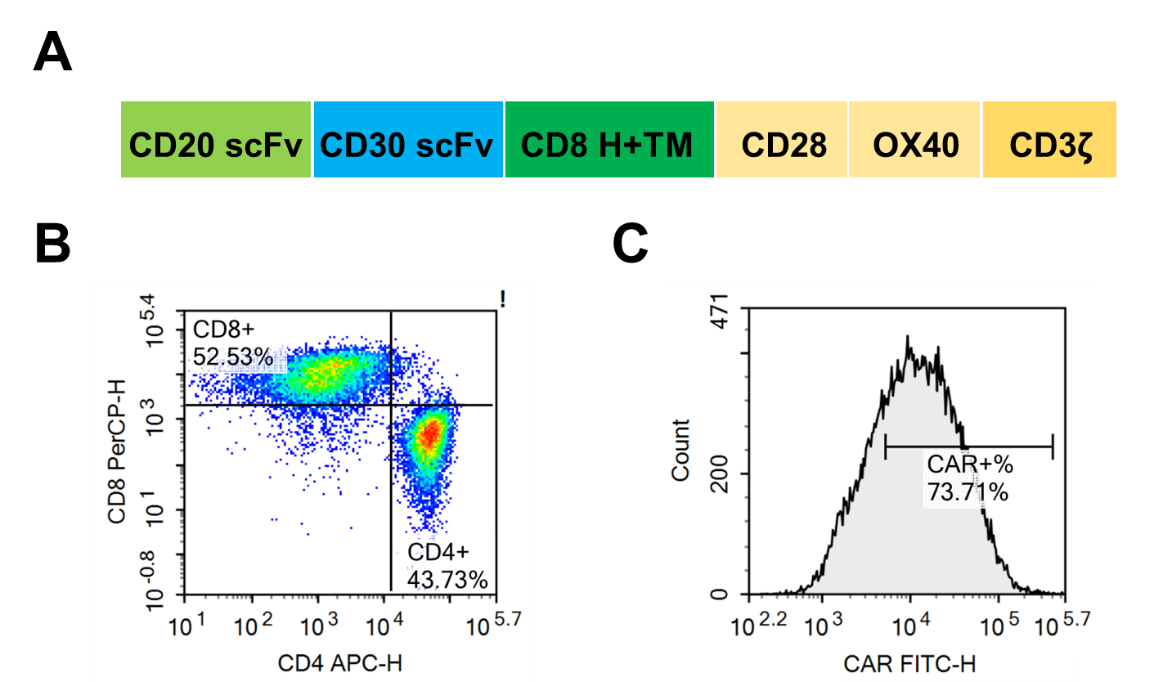


Characterization of anti-CD20/CD30 CAR T-cells product. T cells were selected from patient’s apheresis and transduced with retrovirus encoding bispecific anti-CD20-CD30 CAR post activation. Cells were harvested after 5 days ex vivo expansion and subjected to quality control tests before releasing for infusion.

(A) Schematic of tandem anti-CD20-CD30 CAR construct; scFv, single chain fragment variable; H+TM, hinge and transmembrane domain.

(B-C) Representative flow cytometry dot plots displaying the proportions of CD4+ and CD8+ T cells (B) and CAR+ T cells (C) in manufactured pre-infusion CAR T-cell products.
